# In Vitro and In Vivo Antitumor Activity of Indolo[2,3-*b*] Quinolines, Natural Product Analogs from Neocryptolepine Alkaloid

**DOI:** 10.3390/molecules26030754

**Published:** 2021-02-01

**Authors:** Najla Altwaijry, Samah El-Ghlban, Ibrahim E.-T. El Sayed, Mohamed El-Bahnsawye, Asmaa I. Bayomi, Rehab M. Samaka, Elkhabiry Shaban, Elshaymaa I. Elmongy, Thanaa A. El-Masry, Hytham M. A. Ahmed, Nashwah G. M. Attallah

**Affiliations:** 1 Department of Pharmaceutical Sciences, College of Pharmacy, Princess Nourah bint Abdulrahman University, Riyadh P.O. Box 84428, Saudi Arabia; naaltwaijry@pnu.edu.sa (N.A.); eielmongy@pnu.edu.sa (E.I.E.); Taelmasry@pnu.edu.sa (T.A.E.-M.); ngmohamed@pnu.edu.sa (N.G.M.A.); 2 Department of Chemistry, Faculty of Science, Menoufia University, Shebin El Koom P.O. Box 32511, Egypt; Samah.elghalban@science.menofia.edu.eg (S.E.-G.); m_nh22010@yahoo.com (M.E.-B.); 3 Department of Zoology, Faculty of Science, Menoufia University, Shebin El Koom P.O. Box 32511, Egypt; asmaaibrahiem@science.menofia.edu.eg; 4 Department of Pathology, Faculty of Medicine, Menoufia University, Shebin El Koom P.O. Box 32511, Egypt; rehabsamaka@med.menofia.edu.eg; 5 Dyeing, Printing and Textile Auxiliaries Department, Textile Research Division, National Research Centre, 33 El Bohouth St., Dokki, Giza P.O. Box 12622, Egypt; shaban_nrc@yahoo.com; 6 Department of Pharmaceutical Chemistry, Faculty of Pharmacy, Helwan University, Ain Helwan, Cairo P.O. Box 11795, Egypt; 7 Department of Pharmacology and Toxicology, Faculty of Pharmacy, Tanta University, Tanta P.O. Box 31527, Egypt; 8 Pharmaceutical Analysis Department, Faculty of Pharmacy, Menoufia University, Shebin El Koom P.O. Box 32511, Menoufia, Egypt; hmaahmed@menofia.edu.eg; 9 National Organization of Drug Control and Research (NODCAR), Giza P.O. Box 29 Pyramids, Egypt

**Keywords:** indoloquinoline, cancer cell cycle, apoptosis, antioxidant enzymes

## Abstract

Neocryptolepine (5-methyl-5H-indolo[2,3-b] quinoline) analogs were synthesized and evaluated in vitro and in vivo for their effect versus Ehrlich ascites carcinoma (EAC). The analogs showed stronger cytotoxic activity against EAC cells than the reference drug. The in vivo evaluation of the target compounds against EAC-induced solid tumor in the female albino Swiss mice revealed a remarkable decrease in the tumor volume (TV) and hepatic lipid peroxidation. A noticeable increase of both superoxide dismutase (SOD) and catalase (CAT) levels was reported (*p* < 0.001), which set-forth proof of their antioxidant effect. In addition, the in vitro antioxidant activity of the neocryptolepine analogs was screened out using the DPPH method and showed promising activities activity. The histopathological investigations affirmed that the tested analogs have a remarkable curative effect on solid tumors with minimal side-effect on the liver. The study also includes illustrated mechanism of the antitumor activity at the cell level by flow cytometry. The cell cycle analysis showed that the neocryptolepine analogs extensively increase the aggregation of tumor cells in three phases of the cell cycle (G0/G1, S and G2/M) with the emergence of a hypo-diploid DNA content peak (sub-G1) in the cell cycle experiments, which is a clear-cut for the apoptotic cell population. Furthermore, the immunological study manifested a significant elevation in splenic lymphocyte count (*p* < 0.001) with the elevation of the responsiveness of lymphocytes to phytohemagglutinin (PHA). These results indicate that these naturally-based neocryptolepine alkaloids exhibit marked antitumor activity in vivo and represent an important lead in the development of natural-based anticancer drugs.

## 1. Introduction

**Scheme 1:**
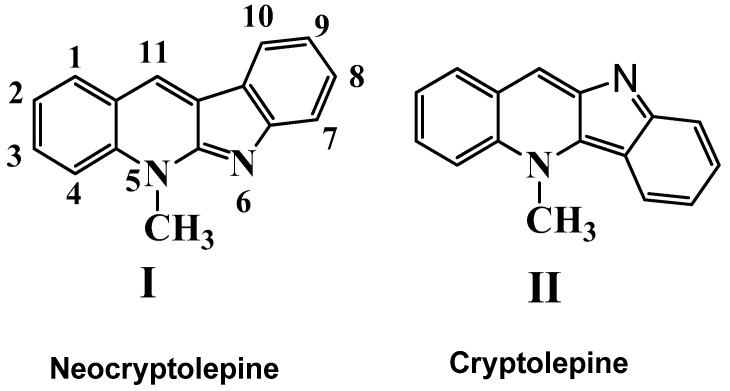
Structure of neocryptolepine **I** and cryptolepine **II** as naturally occurring indoloquinoline alkaloids.

Cancer is one of the biggest concerns in the modern health system. In 2018, 18 million new cancer cases were registered, and 9.5 million people died because of cancer [1]. Cancer is considered one of the major causes of death worldwide, outnumbered only by cardiovascular and infectious diseases. Exploring new drugs to meet the needs of drug-resistant diseases is still a hot topic in medicinal chemistry. Because of the consequence of drug resistance, the present situation is alarming, and new drugs are urgently needed to surmount current issues such as side effects. Meanwhile, nature-inspired products are still important resources for the discovery of new drugs. The potential of natural compounds as new drug leads is clearly illustrated by the discovery and development of taxol, which is isolated from the Pacific yew tree (*Taxus brevifolia*) as a first-line anticancer drug [2,3]. The importance of taxol as an anticancer agent is encouraging researchers around the world to discover another plant-derived substance with anticancer activity. A promising plant appeared to be *Cryptolepis sanguinolenta* [4]. Extracts of the West African shrub *Cryptolepis sanguinolenta* were used for centuries against various disorders, among them malaria, in traditional African medicine. A standardized teabag formulation containing *Cryptolepis sanguinolenta* root powder has been approved recently for the treatment of malaria in Ghana [5]. The biologically active components of the plant extracts are indoloquinoline alkaloids such as neocryptolepine **I** and its regio-isomer, cryptolepine **II** (Scheme 1).

The alkaloid, neocryptolepine **I** or 5-methyl-indolo[3,2-b] quinoline, was isolated recently [6,7,8] showed antibacterial, antischistosomicidal, anticancer and antiplasmodial activity [9,10,11,12,13,14,15,16,17,18,19,20,21,22,23,24,25,26,27,28]. Further experiments indicated that the anticancer activity of indoloquinolines is based on DNA-binding, specifically by DNA base pair intercalation, followed by inhibition of the enzyme topoisomerase II [29]. Some indoloquinolines showed a high-affinity towards triplex and quadruplex DNA [30,31,32,33]. In the search for novel anticancer lead compounds, we focused on neocryptolepine **I,** which also showed anticancer activity against different cell lines.

The aim of this work is to improve the pharmacological profile of the parent natural alkaloid neocryptolepine via chemical modification through the installation of basic amino-substituted side-chains at the C-11 position of the parent compound. A basic side-chain proved to be an important feature for drug activity [29]. The second aim of the current study is to explore how the newly synthesized analogs affect cancer cells to clarify their mechanism of action. In addition, to the best of our knowledge, our study represents the first report on the in vivo antitumor activity of natural-based neocryptolepine analogs.

## 2. Results and Discussion

### 2.1. Chemistry

**Scheme 2:**
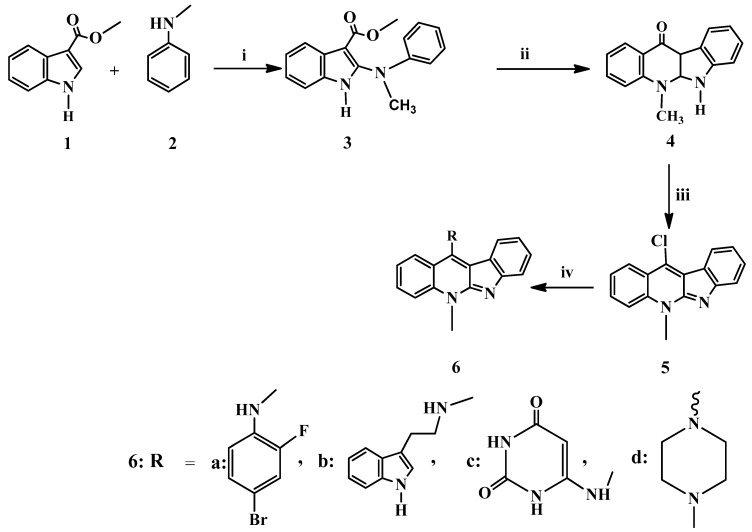
Synthesis of indoloquinolines with 11-substitution.

The target molecules were prepared from methyl-*1H-*indole-*3*-carboxylate **1** and *N*-methylaniline **2**. Intermediate methyl 2-(phenylamino)-1H-indole-3-carboxylates **3** was obtained by chlorination with *N*-chlorosuccinimide in the presence of *1,4-*dimethylpiperazine, followed by the addition of *N*-methyl aniline **2** as trichloroacetate salt. Compounds **3** was further cyclized in boiling diphenyl ether to give 5,6-dihydro-11H-indolo[2,3-b] quinolin-11-one **4,** which was dehydroxychlorinated with POCl_3_ to give up the corresponding *11*-chloroneocryptolepine **5** in good yield as depicted in Scheme 2. The key intermediate **5** was used further for the diversification of the indoloquinoline core (**I**) at the C-11 position. Consequently, 11-chloroneocryptolepine **5** was substituted via S_NAr_ reaction in DMF with the appropriate amines under heating, forming the corresponding 11-aminated products **6a–d** smoothly in good yields as given in Scheme 2.

Reagent and conditions: (i) a. *N*-chlorosuccinimide, 1,4-dimethylpiperazine, CH_2_Cl_2_, 0 °C, 2 h. b. trichloroacetic acid, room temperature, 2 h. (ii) Diphenyl ether, reflux, 3 h. (iii) POCl_3_, toluene, reflux, 12 h. (iv) (1.2 eq) appropriate amine, dry DMF, (10 eqs.) triethylamine, reflux at 135–155 °C for 10–12 h.

The spectroscopic results for all products are in good agreement with the chemical structures and correspond well with the bond formation of the C-11 side-chain nitrogen. In addition, the synthesized neocryptolepine analogs used in biological screening tests have a high HPLC purity ≥94.5%.

### 2.2. Assessment of Cytotoxic Activity In Vitro

The synthesized indoloquinoline analogs **6a–d** were evaluated for their cytotoxic activity against Ehrlich ascites carcinoma (EAC), as depicted in Table 1. The EAC cells used in this study were supplied by National Cancer Institute, Cairo, Egypt. The cells were maintained in vivo in Swiss Albino female mice by intraperitoneal transplantation (0.2 mL of 2 × 10^6^ cells/mL). EAC cells were aspirated from the peritoneal cavity of mice, washed with saline and given intraperitoneally to develop ascites tumor. Trypan blue dye exclusion technique [34] was used to figure the viability percentage of EAC cells to assay the cytotoxic activity of the studied compounds. In brief, under completely sterile conditions, EAC cells were separated away from the mice abdomens. Hank’s balanced salt solution (HBSS) was used to wash the separated cells. Cells were put in a cooling centrifuge at 1500 rpm for 15 min; the produced granules were put back into suspension using HBSS, the mentioned steps were done in triplicate. Finally, a measured volume of RPMI 1640 was used to suspend the cells, followed by the addition of 10% fetal bovine serum, 10 µg/mL streptomycin and 100 U/mL penicillin. Consequently, the cell count was adjusted to 2 × 106 cells/mL from the prepared diluted cell suspension 0.2 mL was titrated in 96 flat-bottomed tissue culture plates. Serial concentrations of each tested compound were prepared and added in triplicate and incubated at 37 °C for 3 h in a 5% CO_2_ atmosphere. The IC50 parameter (half-maximum inhibitory concentration) was calculated using Trypan blue dye exclusion test, and then the cytotoxic effect was evaluated using control EAC cells [34]. Thalidomide was used as a reference drug alongside the parent natural compound neocryptolepine I for comparison. Thalidomide was used as a reference drug because its mechanism of action is expected to be similar to the mechanism of action of neocryptolepine analogs as its anticancer action may be based on its antiangiogenic action, resulting from specific DNA intercalation as well as induce cell cycle arrest [34,35].

The percentage of EAC cell death of the synthesized derivatives was evaluated by trypan blue exclusion assay [34]. In addition, the compounds 6a–d, as well as the reference drug thalidomide and the parent natural compound neocryptolepine (I), were evaluated through eight concentration levels. It is worth noting that this cytotoxicity was dose-dependent when compared to the reference drug, thalidomide and the control, which was treated with the corresponding dilutions of DMSO. The analogs 6b and 6d were the highest cytotoxic against Ehrlich ascites carcinoma with IC_50_ 6.4 × 10^−5^ and 1.5 × 10^−4^ µM, respectively and exhibited higher antiproliferative activities than the reference drug, thalidomide (IC_50_ = 2.6 × 10^−4^ µM) as well as the parent natural compound I (IC_50_ = 5.4 × 10^−4^ µM). Based on these results, the neocryptolepine analogs represent a promising target for further in vivo study.

### 2.3. In Vitro Antioxidant Activity of Indoloquinoline Derivatives

Over-production and the accumulation of free radicals inside the body leads to a phenomenon known as oxidative stress, which causes many serious diseases such as cancer, cardiovascular, and inflammation. Moreover, the harmful effects of this phenomenon can be reduced via using antioxidant compounds as free radical scavengers [36,37,38,39]. Therefore, free radical scavenging capabilities were determined for all compounds by 2,2-diphenyl-1-picrylhydrazyl (DPPH) assay using ascorbic acid (AA) as standard for comparison. The DPPH reducing capabilities of the synthetic analogs **6a–d** were evaluated by determining their IC_50_ values. The antioxidant activity analysis of the indoloquinones **6b** and **6d** indicated that strong reducing activity and inhibitory effects on superoxide radical formation. The IC_50_ values of all compounds obtained in the DPPH assay are presented in Table 2. The IC_50_ value corresponds to the concentration of a sample, which has the ability to scavenge 50% of the free radicals present in the reaction mixture. Low IC_50_ values indicate that the compound in the sample has high antioxidant activity. As shown in Table 2, all compounds had free radical scavenging activity in the DPPH assay. The IC_50_ values of all four indoloquinolines in the DPPH assays were in the range of 3.3–9.2 × 10^−5^ µM and had comparable DPPH free radical scavenging activity to the standard ascorbic acid with IC_50_ 4.2 × 10^−5^ µM. Compound **6d**, which has *N*-methylpiperazine moiety, showed the highest antioxidant activity with IC_50_:3.3 × 10^−5^ µM and can be considered as the most active one among the four analogs. At the same time, the analog **6c** with pyrimidine moiety had the lowest antioxidant activity (9.2 × 10^−5^ µM). The differences in free radical scavenging activity among the 4 compounds could be related to the presence of different characteristic side-chain groups at C-11 of the indoloquinoline core structure. In addition, the parent compound, neocryptolepine is shown lower antioxidant activity with IC_50_:1.6 × 10^−5^ µM, which is considered lower than the 11-substituted analogs. This indicating the important feature of the side-chain for the antioxidant activity of the parent natural compound neocryptolepine-(I) (cf. Scheme 1).

### 2.4. Short-Term Toxicity Study

To confirm the safety of indoloquinoline compounds, acute and short-term toxicity studies were done. Forty-two Swiss albino mice were divided into six groups of seven mice each. The first five groups were subcutaneously injected in single graded doses. The first five groups were given a single dose of graded doses (400, 800, 1200, 1600, and 2000 mg/kg body weight) of indoloquinoline analog containing 11-substituted N-methyl piperazinyl side-chain **6d** by the subcutaneous route, while the last group received 0.2 mL of (water: DMSO, 7:3) by the same route. The animals were then observed for 24 h for toxicity signs and death. The LD_50_ of **6d** was calculated using the arithmetic methods of Karber as modified by Aliu and Nwude [40]. The mortality rates and calculated LD_50_ are presented in (Table 3). The result of the subcutaneous acute toxicity study showed that LD_50_ of **6d** is 1000 mg/kg, which indicates that **6d** could be considered as a low toxicity compound was higher safety margins.

### 2.5. Antitumor Activity: Percentage Reduction in Tumor Volume

The aim of this study is to characterize indoloquinoline analogs **6a–d** curative ability and antineoplastic activity on solid-form of Ehrlich tumors. Tumor volume is a potential biomarker for Ehrlich tumor development. The induction of solid tumor in Swiss female mice performed by inserting 0.2 mL of EAC cells (2 × 10^6^ cell/mL) subcutaneously (SC) between thighs of the lower limb. When the tumor volume reached 15–20 mm^3^ (Seven days after tumor induction), the mice were randomly divided into 6 groups (*n* = 10), five groups were injected subcutaneously with thalidomide and indoloquinoline analogs **6a–d** (100 mg/kg) daily for five consecutive days, the sixth group (positive control group) were given DMSO/saline (0.3/0.7). The 8th day is considered the zero days of treatment. The tumor volume was daily measured with a vernier caliper for four days stated from day eight.

**Figure 1:**
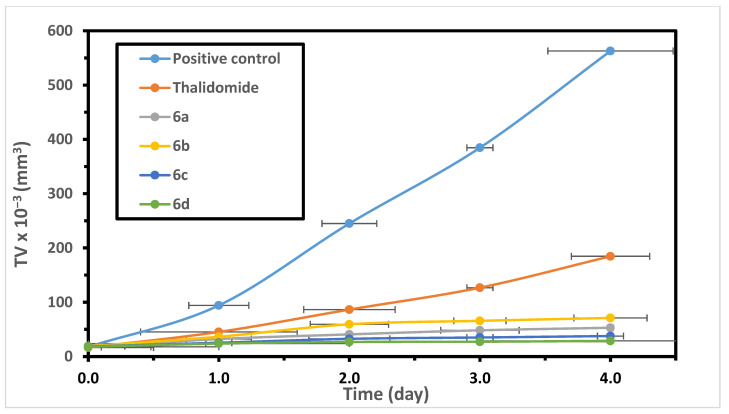
Effect of thalidomide and indoloquinoline analogs **6a**, **6b**, **6c** and **6d** on tumor volume changes of the six groups over the course of the treatment. Values expressed as means ± SD.

All the treatments showed a significant (*p* < 0.001) reduction in tumor volume as compared to the positive control. The maximum reduction in tumor volume was seen in treatment groups with **6a–d** compared with positive control and thalidomide groups. Treatment with compounds **6a–d** showed a significant (*p* < 0.001) reduction in tumor volume as compared to positive control and thalidomide groups. Thalidomide showed a significant (*p* < 0.001) reduction percentage in tumor volume as compared to the positive control, but lower than 6a–d. The reduction percentage in tumor volume on day 12 (the last day of treatment) was 67.20 for reference drug (Thalidomide), 90.58, 87.37, 93.29 and 94.90% for **6a, 6b, 6c** and **6d**, respectively, which were significantly (*p* < 0.001) high compared to control and thalidomide groups (Figure 1). The obtained results suggested that the newly synthesized indoloquinoline derivatives establish an encouraging base for searching for new structure optimization to acquire safe and effective antitumor drug agents.

### 2.6. Effect of Indoloquinoline Derivatives on Lipid Peroxidation and Antioxidant Enzymes

Measuring antioxidant enzyme levels and lipid peroxidation was utilized to evaluate the pharmacological effect of indoloquinoline derivatives. The study group was divided into two subgroups, normal mice and tumor-bearing mice. Each subgroup is further subdivided into two groups, one group received indoloquinoline derivatives, and the other group was marked as a placebo group.

Study results showed that normal mice that have been given indoloquinoline derivatives at a dose of 100 mg/kg for 5 constitutive days subcutaneously showed no significant changes in the levels of lipid peroxidation and antioxidant enzymes over their normal counterparts (Table 4).

A group of solid-tumor bearing mice was treated with 100 mg/kg body weights with thalidomide and the indoloquinoline analogs **6a, 6b, 6c** and **6d** for five constitutive days, Table 5, shows the changes in the level of malondialdehyde (MDA), malondialdehyde is the end product of lipid peroxidation and antioxidant enzymes activities. The obtained results revealed that the liver tissue of the positive control group reported a remarkable increase in the levels of lipid peroxidation in comparison with the normal group (*p* < 0.001), as stated in (Table 5). After administration of indoloquinoline analogs **6a, 6b, 6c** and **6d** at a dose of 100 mg/kg for 5 constitutive days to mice with EAC, the levels of lipid peroxidation were highly significant less than that of the positive control group (*p* < 0.001). Meanwhile, the level of SOD in the liver homogenate of positive bearing mice was 32.1% (*p* < 0.001) less than that of the normal group (Table 5). Post-treatment with the tested compounds **6a, 6b, 6c** and **6d** at a dose 100 mg/kg for five constitutive days, levels of SOD showed a very highly significant increase by 54.4%, 53.69, 36.24% and 39.8%, respectively, in relation to that of the positive control group (*p* < 0.001). Similar to SOD, the catalase (CAT) level in the positive control group showed a significant decrease (41.8%-*p* < 0.001) up against the normal group (Table 5). After giving compounds **6a**, **6b**, **6c** and **6d**, CAT levels were remarkably increased by 51.2%, 78.04%, 107.3% and 65.8%, respectively, when measured against that of positive control (*p* < 0.001). These findings suggest that these compounds may exert their antitumor activity via two pathways:

1. Reducing the peroxidation of lipids by scavenging the free radicals;
2. By increasing the level of antioxidant enzymes.

### 2.7. Effect of Indoloquinoline Derivatives on Splenic Lymphocyte Count

The difference in the number of splenic lymphocytes between the study groups, including groups given indoloquinoline derivatives, control group, and groups with a tumor, were evaluated. Normal mice administered indoloquinoline derivatives at a dose of 100 mg/kg for 5 constitutive days subcutaneously reported statistical similarities with the untreated normal mice group regarding the count of splenic lymphocytes (Table 6).

These results proved that the used dose of indoloquinoline derivatives is not immunotoxic itself. In this study, the group with EAC showed a marked reduction in splenic lymphocytes. This reduction is reconfirming the reported fact that tumor causes massive depletion in immune cell numbers [41]. Table 7 shown the reduced splenic lymphocyte results were a reaffirmation that cancer disabled normal lymphocyte development. The effective dose of indoloquinoline analogs, given throughout this study; 100 mg/kg for 5 constitutive days subcutaneously, reported an increase in the spleen cell number providing evidence that they protect the lymphoid organ of mice with tumor as illustrated in (Table 7). The group of EAC-bearing mice that received 100 mg/kg of indoloquinoline analogs **6a, 6b, 6c** and **6d** for 5 constitutive days subcutaneously reported remarkable increase (*p* < 0.001) in lymphocytic count (440.5%, 300%, 359.5% and 359.5%, respectively).

### 2.8. Lymphoproliferative Response to Phytohemagglutinin

Table 8 shows the lymphoproliferative response to phytohemagglutinin (PHA) mitogen, expressed as count per minute (cpm) of tested groups of the study. Results revealed an appreciable reduction (*p* < 0.001) in the response of lymphocytes to PHA in the positive control group relative to that of the normal control group as listed in (Table 8). Moreover, a remarkable increase (*p* < 0.001) in the activity of the lymphocytes was reported after giving indoloquinoline derivatives to mice bearing EAC relative to the positive control group, as shown in (Table 8).

Normal mice that have been given indoloquinoline derivatives at a dose of 100 mg/kg for 5 constitutive days subcutaneously showed no significant changes in the response of lymphocytes to PHA relative to the normal group as illustrated in Table 9.

### 2.9. Effect of Indoloquinoline Analogs on Cell Cycle of EAC Cell

Flow cytometry is a technique that gives a clue to the mechanism by which cytotoxic agents affect the cell cycle by sorting and analyzing cells based on their content of DNA. Flow cytometry was used to further evaluate the effects of the indoloquinoline compounds modes-of-action to the cellular level, and we extended our studies on the cell cycle effects. To know whether cell cycle changes were aligned with the inhibition of cell growth, the cell cycle phase distribution was examined via flow cytometry. The cell cycle was analyzed in EAC-bearing mice treated with indoloquinoline analogs **6a, 6b, 6c** and **6d** (at a dose of 100 mg/kg for 5 constitutive days).

**Figure 2:**
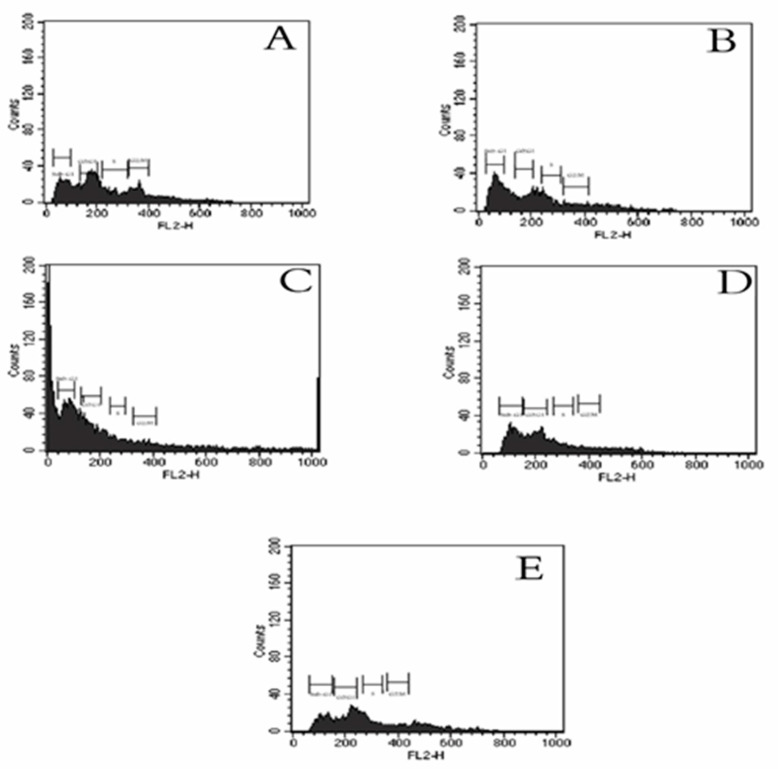
Flow-cytometric analysis of EAC cell cycle phase distribution. Cells from EAC bearing mice treated with 0.3 DMSO (positive control) and EAC bearing mice treated with 100 mg/kg for 5 constitutive days subcutaneously of indoloquinoline analogs **6a, 6b, 6c** and **6d**, fixed and nuclear DNA was labeled with PI. Cell cycle phase distribution of EAC nuclear DNA was determined by single label flow-cytometry. Histograms display of DNA content (*x*-axis, PI-fluorescence) versus counts (*y*-axis) showed (**A**) EAC of positive control, (**B**) EAC of **6a**-treated EAC bearing mice, (**C**) EAC of **6b**-treated EAC bearing mice, (**D**) EAC of **6c**-treated EAC bearing mice; (**E**) EAC of **6d**-treated EAC bearing mice.

Flow cytometry was used to calculate the percentage of cells in each phase of the cell cycle in both positive control (exposed to 0.3% DMSO) and treated cells, results illustrated in (Table 10 and Figure 2). The influence of indoloquinoline derivatives on EAC cells indicated noticeable cell cycle specificity. In comparison with the positive control group, an appreciable change in the cell cycle occurred in giving the studied compounds. The sub-G1 population indicated apoptotic-associated chromatin degradation. As compared to control, the sub-G1 group increased by 106%, 1452%, 683%, and 975% after treatment with compound **6a**, **6b**, **6c** and **6d,** respectively (*p* < 0.05) and (*p* < 0.001). In the non-apoptotic population, compound **8a** increased the G0/G1 cell population by 17.4%, while it reduced the *S*-phase fraction by about 35.3% and the G2/M fraction by 69.7% relative to positive control cells (*p* < 0.05). Compound **6b** increased the G0/G1 cell count by 2%, while it reduced the *S*-phase fraction by about 70.3% and increased the G2/M fraction by 26.2% relative to control cells (*p* < 0.05). Compound **6c** produces a reduction in the cell fraction went into the G0/G1 phase by about 95.7%, at the same time, an approximate increase of 411.3% and 269.5% of cells in the S-phase and the G2/M fraction, respectively, were reported relative to positive control cells (*p* < 0.05). Compound 6d reduced the G0/G1 and S-phase fractions by 46% and 56.6%, respectively (*p* < 0.05), at the same time it increased the G2/M fraction by 263.3% (*p* < 0.001).

In summary, it is clear that compound **6a**–**d** exerted its antitumor activity via inhibiting proliferation of the cells, which resulted in seizing of the cell cycle at G0/G1, S, G2/M phases leading to an induction of apoptosis. Consequently, the visibility of “sub-G1” peaks by flow-cytometry may be a function of individual cellular homeostasis.

It has been reported that thalidomide induced cell cycle arrest by increasing the number of cells in the G0/G1 phase and decreasing the percentage of S phase in MG-63 cells [34,35].

### 2.10. Histopathological Examination

**Figure 3:**
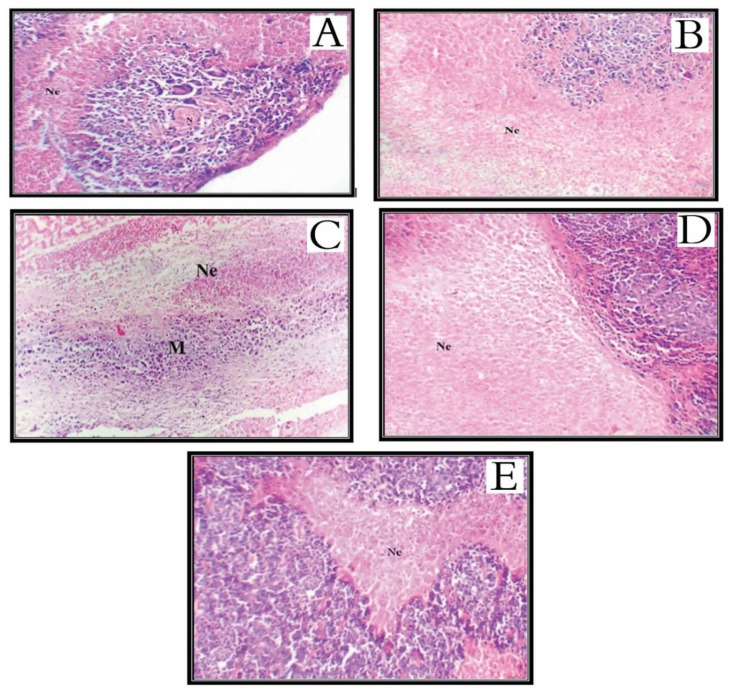
(**A**) Tumor section of Ehrlich positive control mouse showed an area of necrosis (Ne). (**B**) Tumor section of Ehrlich ascites carcinoma (EAC) bearing mouse treated with compound **6a** displayed massive, coagulated necrosis (Ne) with minimal residual malignant cells at the upper right corner. (**C**) Tumor section of Ehrlich ascites carcinoma (EAC) bearing mouse treated with compound **6b** displayed extensive necrosis (Ne) with minimal viable malignant cells. (**D**) Tumor section of Ehrlich ascites carcinoma (EAC) bearing mouse treated with compound **6c** showed a massive area of coagulative necrosis (Ne) with viable tumor cells at the right side. (**E**) Tumor section of Ehrlich ascites carcinoma (EAC) bearing mouse treated with compound **6d** showed massive geographic coagulative necrosis (Ne) surrounded by viable tumor cells. (H and E × 200).

Results illustrated in (Figure 3) showed an extensive cell death rate of the solid tumor on using indoloquinoline compounds **6a** to **6d** as compared to the positive control experiments. Whereas indoloquinoline analogs **6a** and **6b** reported supremely cell death percentage and apoptosis relative to that of other molecules, **6c** and **6d**.

An essential pathway in fighting tumors is apoptosis; in the present study tumor-bearing mice group which received indoloquinoline analogs **6a, 6b, 6c** and **6d** exhibited raise in the number of apoptotic cells indicated by histopathological examination of H and E staining.

**Figure 4:**
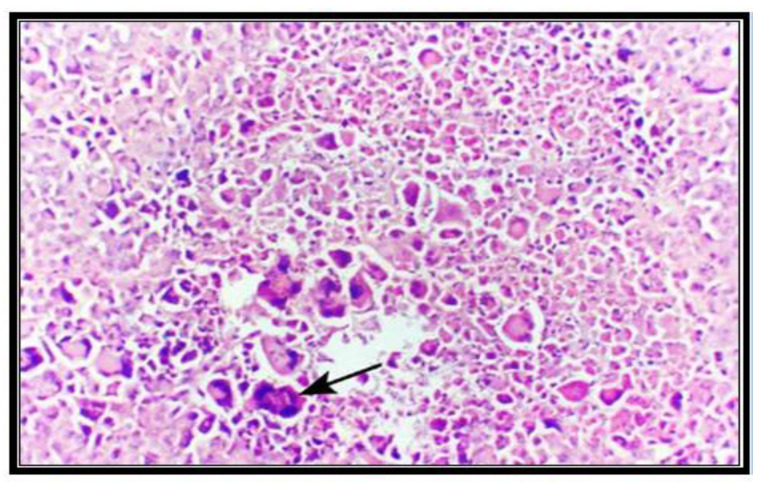
Malignant tumor of Ehrlich ascites carcinoma (EAC) bearing mouse treated with indoloquinoline compounds **6a**, **6b**, **6c** and **6d** showed criteria of anaplasia, necrosis. Moreover, small clusters of apoptotic cells (arrow) were observed. The apoptotic cells displayed dense basophilic nuclei and dense eosinophilic cytoplasm. (H and E × 200).

Results of the treated group and that of the positive control group are illustrated in (Figure 3 and Figure 4). Treatment with compounds **6a** and **6c** displayed the strongest apoptotic activity over the rest of compounds **6b** and **6d**. At the same time, the count of mitotic cells was less in the studied analogs **6a, 6b, 6c** and **6d** as measured up to that of the positive control group. The greatest antimitotic effect was given by molecule **6a**.

**Figure 5:**
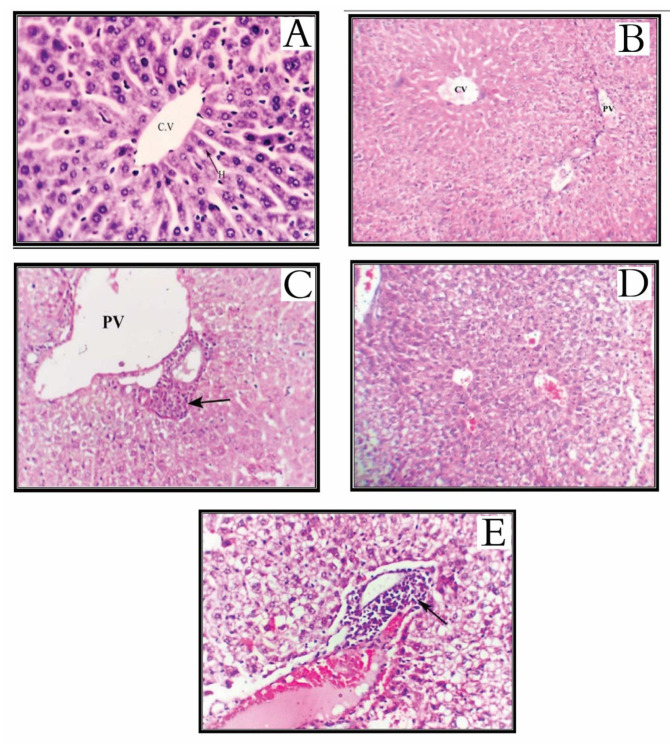
(**A**) Liver section of normal mouse showing cords of hepatocytes (H) radiating from the central vein (C.V) and separated by blood sinusoids (H and E × 400). (**B**) Liver section of normal mouse treated with compound **6a** showed preserved lobular architecture with mild degenerative changes. Central vein (CV) and portal vein (PV) could be seen. (**C**) Liver section of a normal mouse treated with compound **6a** showed mild degenerative changes and mild portal lymphocytic infiltrates (arrow) surrounding the portal vein (PV). (**D**) Liver section of normal mouse treated with compound **6b** showed mild diffuse degenerative changes, mild congestion, and scattered lymphocytes. (**E**) Liver section of normal mouse treated with compound **6c** showed a moderate diffuse degenerative change in the form of feathery degenerative changes. Moreover, a wide dilated portal vein and mild portal perivascular lymphocytic infiltrates (arrow) are seen. (×200 H and E).

It is well known that the vital organ enables the human body to get rid of toxins, as organic xenobiotics in the liver. Tissue changes in the liver are linked with histological abnormalities. Possible unwanted effects of the Indoloquinolines were checked in the mice group that received the compound; 5 days after subcutaneous injection of the compounds, tumors and livers were fixed in 10% neutral buffered formalin solution and embedded in paraffin. Sections, 8 m thick, were stained using hematoxylin and eosin (H and E). The indoloquinoline analogs **6a**, **6b**, **6c** and **6d** did not cause necrosis to liver cells (Figure 5). Compound **6b** gave the highest effect in tumor cell death without harming liver cells in comparison to other molecules, while treatment with compound **6c** showed remarkable liver degeneration.

## 3. Materials and Methods

### 3.1. Chemistry

#### 3.1.1. General

Starting materials were either commercially available or prepared. Anhydrous solvents used in this study were obtained from Sigma-Aldrich. The purity of the compounds was verified by TLC on silica gel 60 F254 (Merck). ^1^H- and ^13^C-NMR spectra were recorded in CDCl_3_ or DMSO-d6 on a Bruker DRX-600 spectrometer operating at 600 MHz for ^1^H analyses and 150 MHz for ^13^C analyses. Chemical shift data are expressed in ppm with reference to TMS. IR spectra are recorded on a Perkin-Elmer 1600 FTIR. Melting points were determined on a Kofler apparatus and were not corrected. The anticancer activity was executed at the Zoology Department, Faculty of Science, Menoufia University, Egypt. EAC cells were supplied by National Cancer Institute, Cairo University, Egypt, Hank’s balanced salt solution (HBSS), RPMI 1640 (SIGMA Aldrich, St Louis, MO, USA), 3. 2,2′-diphenyl-1-picryl-hydrazyl (DPPH) (Sigma Aldrich, Saint-Louis, MS, USA), Swiss female albino mice (Theodor Bilharz Research Institute (TBRI), Giza, Egypt), Swiss female albino mice of CD-1 strain (weighing 22 ± 2 gm at the beginning of the experiments) were obtained originally from Schistosome Biological Supply Program (SBSP) unit at Theodor Bilharz Research Institute (TBRI) Giza, Egypt. Phytohemagglutinin (PHA) (Sigma-Aldrich), [3H] thymidine (Sigma-Aldrich), flow cytometer (Becton Dickinson, Sunnyvale, CA, USA), hematoxylin, and eosin (H and E) (Sigma-Aldrich). The flow cytometer was carried out with FACS caliber flow cytometer (Becton Dickinson, Sunnyvale, CA, USA) equipped with a compact air cocked low-power 15 m watt argon ion laser beam (488 nm). Data analysis was conducted using the DNA analysis program MPDFIT. This software calculated the CV around the G0/G1 peak and the percentage of cells in each phase (G0/G1, S and G2/M) of the DNA cell cycle for each sample. Purity was verified using HPLC AND performed on Agilent 1100 Series integrated system equipped with aG1313A automated injector, aG1311A quaternary pump and G1315B diode-array detector (DAD). The chromatographic separation of the compounds was achieved with a reversed-phase column, ZOBRAX XDB-C18 (5.0 × 150.5 µm) from Agilent (USA) operating at a constant flow rate of 1 mL/min and temperature (20 °C). The chromatographic data were analyzed using Agilent Chemstation B.02.01.LC–MS Spectra were recorded on an Agilent 1100 series HPLC system using Alltech Prevail C18 column (2.1 mm × 50 mm, 3 µm) coupled with an Esquire 3000plus as MS detector and 5–100% B, 20 min-gradient was used with a flow rate of 0.2 mL/min. Then 0.1% formic acid was added to solvent A and B. LC-UPLC-ESI-MS positive and negative ion acquisition mode was carried out on a XEVO TQD triple, quadruple system. Waters Corporation, Milford, MA01757 USA, mass spectrometer column: ACQUITY UPLC-BEH C18 1.7–2.1 × 50 mm column, flow rate: 0.2 mL/min, solvent system: consisted of (A) water containing 0.1% formic acid (B) acetonitrile.

#### 3.1.2. 2-(Methylphenylamino)-1H-indole-3-carboxylic Acid Methyl-Ester (3)

Methyl 1*H*-indole-3-carboxylate **(1)** (2.25 g, 12.9 mmol) was dissolved in CH_2_Cl_2_ (53 mL) in a 250 mL round-bottomed flask at 0 °C under nitrogen. 1,4-Dimethylpiperazine (0.9 mL, 6.6 mmol) and *N*-chlorosuccinimide (2.25 g, 16.8 mmol) were added. The reaction mixture could stand at 0 °C for 2 h, and a solution of trichloroacetic acid (0.57 g, 3.37 mmol) and N-methyl aniline (0.96 mL, 8.92 mmol) in CH_2_Cl_2_ (53 mL) was added, and the reaction mixture was allowed to attain room temperature for two hours. A washing sequence of 10% aqueous sodium bicarbonate solution followed by 1 M aqueous hydrochloric acid, and finally, water was applied to the mixture. To obtain the formed product, residue steps of drying, filtration and evaporation were performed for the resulting solution. The residue was chromatographed using hexane/ethyl acetate 8:2 as eluent as to yield white powder (3.3 g, 91%), m.p. 146–147 °C, ^1^H NMR (CDCl_3,_ 600 MHz) δ 3.35 (s, 3H, N-CH_3_), 3.65 (s, 3H, O-CH_3_), 6.70–6.80 (m, 2H, Ar-H), 6.82–6.85 (m, 1H, Ar-H), 7.15–7.33 (m, 4H, Ar-H), 7.33 (m, 1H, Ar-H), 11.97 (s, 1H, NH). ^13^C NMR (CDCl_3_, 150 MHz): δ 41.0, 51.8, 97.8, 111.2, 115.5, 120.4, 121.7, 122.3, 125.5, 128.9, 131.9, 146.4, 148.1, 165.8.

#### 3.1.3. 5-Methyl-5,6-dihydro-5H-indolo[2,3-b] Quinolin-11-one (4)

The ester **3** (2.02 g, 7.2 mmol) in diphenyl ether (13 mL) was refluxed at (250 °C) for 2 h. Brown solid was formed after cooling the reaction mixture to room temperature and collected by filtration then washed with diethyl ether three times (3 × 30 mL) then dried under vacuum to afford **4** as a gray solid. Yield (1.36 g, 88%), m.p. > 360 °C, ^1^H NMR (DMSO-d_6_, 600 MHz): δ 3.98 (s, 3H, N-CH_3_), 7.25 (m, 2H, Ar-H), 7.42 (m, J = 7.9 Hz, 1H, Ar-H), 7.45 (m, J = 7.0 Hz, 1H, Ar-H), 7.73 (m, 2H, Ar-H), 8.19 (d, J = 7.0 Hz, 1H, Ar-H), 8.39 (d, J = 7.0 Hz, 1H, Ar-H), 12.07 (s, 1H, NH). ^13^C NMR (CDCl_3_, 150 MHz): δ 35.2, 103.6, 112.8, 116.5, 120.3, 121.3, 121.7, 122.3, 124.1, 124.8, 126.7, 132.2, 134.9, 138.8, 145.9, 172.3.

#### 3.1.4. 11-Chloro-5-methyl-5H–indolo[2,3-b] Quinoline (5)

Compound **4** (1 g, 4 mmol) was suspended in dry toluene (5 mL) then POCl_3_ (3 mL, 35 mmol) was added. The reaction mixture was refluxed overnight, cooled to room temperature, then poured into ice and basified with a cold saturated solution of NaHCO_3_ while keeping the internal temperature below 30 °C. The water layer was extracted with dichloromethane (3 × 30 mL). The combined organic layer was washed with water and brine, dried by anhydrous Na_2_SO_4_, and then evaporated under reduced pressure to yield the crude product **5**. The crude product **5** was further purified by column chromatography using EtOAc/hexane (1:1) to afford the pure **5** as orange solid. Yield (1 g, 92%), m.p. 310–312 °C, ^1^H NMR (CDCl_3_, 600 MHz): δ 4.34 (s, 3H, N-CH_3_), 7.30 (m, 1H, Ar-H), 7.51 (m, 1H, Ar-H), 7.73 (m, 3H, Ar-H), 8.36 (d, J = 8.4 Hz, 1H, Ar-H), 8.44 (m, 1H, Ar-H), 8.87 (d, J = 8.4 Hz, 1H, Ar-H). ^13^C NMR (CDCl_3_, 150 MHz): δ 36.1, 113.8, 117.5, 119.6, 120.7, 122.3, 123.3, 123.8, 124.7, 126.4, 129.3, 133.1, 134.9, 136.8, 155.5, 157.3.

#### 3.1.5. N-(4-Bromo-2-fluorophenyl)-5-methyl-5H-indolo[2,3-b] Quinolin-11-amine (6a)

Yield: 0.09 g (61%); mp: 178–180 °C; IR (KBr) v max: 3391, 1593, 1540, 1514, 1385, 1247, 1158, 1099, 897, 858, 746, 721, 615 cm^−1^. ^1^H (CDCl_3_, 600 MHz): δ 4.26 (s, 3H, N–CH_3_), 6.44 (d, 1H, J = 8 Hz), 6.81 (m, 1H), 6.93 (m, 2H, 1H), 7.18 (m, 2H), 7.65 (d, 1H, J = 8 Hz), 7.65 (m, 1H), 7.89 (m, 1H), 8.16 (d, 1H, J = 8.8 Hz), 8.79 (d, 1H, J = 8 Hz), 10.20 (br s,1H). ^13^C NMR (CDCl_3_, 150 MHz): δ 33.37, 112.54, 114.23, 114.48, 114.30, 116.24, 117.35 (2C), 119.31, 119.43, 120.82, 122.46, 124.03, 124. 37, 125.18, 130.86, 133.91, 136.11, 144.71, 145.5, 154.24, 156.30. HPLC purity 96%. LC–MS (ESI), *m*/*z* Calcd for C_22_H_15_BrFN_3_ Exact mass: 419.04, found 420.0848 [M + H]^+^.

#### 3.1.6. N-(2-(1H-Indol-3-yl) ethyl)-5-methyl-5H-indolo[2,3-b] Quinolin-11-amine (6b)

Yield 0.14 (90%); mp: 195–197 °C; IR (KBr) v max: 3344, 3284, 3092, 2954, 2923, 2864, 1646, 1610, 1541, 1512, 1499, 1441, 1418, 1384, 1243, 1248, 1071, 887, 740 cm^−1^. ^1^H NMR (CDCl_3_, 600 MHz): δ 3.32 (t, 2H, J = 6.18 Hz,–CH_2_–CH_2_N), 4.09–4.15 (m, 2H), 4.20 (s, 3H, N–CH_3_); 7.12–7.98 (m, 13H, Ar–H); 8.41 (s,1H, NH). 10.80 (br s, 1H); ^13^C NMR (CDCl_3_, 150 MHz,): δ 32.45, 38.98, 49.64, 111.21, 112.5, 114.04, 117.69, 117.99, 118.23, 119.79, 120.69, 120.77, 120.94, 121.78, 122.46, 123.98, 127.26, 127.91, 128.19, 129.26, 129.89, 130.27, 136.19, 136.96, 155.50, 156.24. HPLC purity 94.5%. LC–MS (ESI), *m*/*z* Calcd for C_26_H_22_N_4_; Exact mass: 390.18, found 391.0817 [M + H]^+^.

#### 3.1.7. 5(5-Methyl-5H-indolo[2,3-b] quinolin-11-ylamino) Pyrimidine-2,4(1H,3H)-dione (6c)

Yield 0.072 (54%); mp: 185–187 °C; IR (KBr) v max: 3273, 3196, 3068, 1698, 1681, 1623 cm^−1^. ^1^H NMR (CDCl_3_, 600 MHz): δ 4.33 (s, 3H, N–CH_3_), 7.70–6.80 (m, 6H, C_6_H_4_), 8.40–7.71 (m, 2H, C_6_H_4_), 10.56 (s, 1H, NH), 10.95 (s, 1H, NH), 11.15 (s, 1H, NH), ^13^C NMR (CDCl_3_, 150 MHz) δ: 32.25, 112.22, 119.30, 120.10, 123.20, 124.30, 125.41, 126.6, 126.9, 128.18, 129.33, 129.62, 131.3, 136.19, 140.2, 147.23, 150.23, 151.30, 151.91, 161.28. HPLC purity 94%. LC–MS (ESI), *m*/*z* Calcd for C_20_H_15_N_5_O_2_; Exact mass: 357.12, found 357.3699 [M]^+^.

#### 3.1.8. 5-Methyl-N-(4-methylpiperazin-1-yl)-5H-indolo[2,3-b] Quinolin-11-amine (6d)

Yield: 0.074 g (66%); mp: 139–141 °C; IR (KBr) v max: 3377, 2985, 2867, 1650, 1608, 1574, 1558, 1493, 1435, 1444, 1202, 1100, 868, 758 cm^−1^. ^1^H NMR (CDCl_3_, 600 MHz): δ 2.62 (s, 3H, N–CH_3_); 2.81 (t, 4H, J = 4.8 Hz, 2CH_2_N), 3.61 (t, 4H, J = 4.8 Hz, 2CH_2_N), 4.35 (s, 3H, N–CH_3_); 7.05–7.43 (m, 7H, Ar–H), 8.93 (s, 1H, Ar–H). ^13^C NMR (CDCl_3_, 150 MHz) δ: 35.10, 32.94 (2C), 52.9 (2C), 53.2, 114.04, 117.69, 119.79, 120.77, 120.94, 121.78, 123.98, 127.91, 128.19, 129.26, 129.89, 130.27, 136.96, 155.50, 156.24. HPLC purity 99%. LC–MS (ESI), *m*/*z* Calcd for C_21_H_20_N_4_; Exact mass: 330.18, found 329.012 [M − H]^−^.

### 3.2. Cytotoxic Activity

#### 3.2.1. Tumor Cells

The supplier of EAC cells was the National Cancer Institute, Cairo, Egypt. The cells were relocated in the peritoneum of Swiss albino female mice (0.2 mL of 2 × 10^6^ cells/mL). EAC cells were separated from the peritoneal cavity of mice, saline washed and injected intraperitoneally to grow ascites tumor.

#### 3.2.2. In Vitro Cancer Screening, Preparations, and Methodology

As a preparation step to perform cytotoxicity assay against the EAC cell line, thalidomide, parent neocryptolepine and the indoloquinoline compounds a **6a**, **6b**, **6c** and **6d** were dissolved in DMSO/saline (0.3:0.7) to prepare a solution of 10 mmol concentration. The stock solutions were stored at 4 °C and were used all over the assay. EAC cells were supplied by the National Cancer Institute, Cairo, Egypt. The cells were maintained in vivo in Swiss albino female mice by intraperitoneal transplantation (0.2 mL of 2 × 10^6^ cells/mL). EAC cells were aspirated from the peritoneal cavity of mice, washed with saline, and given intraperitoneally to develop ascites tumor.

Trypan blue dye exclusion technique [34] was used to figure the viability percentage of EAC cells to assay the cytotoxic activity of the studied compounds. In brief, under completely sterile conditions, EAC cells were separated away from the mice’s abdomen. Hank’s balanced salt solution (HBSS) was used to wash the separated cells. The cells were put in a cooling centrifuge at 1500 rpm for 15 min; the produced granules were put back into suspension using HBSS, the mentioned steps were done in triplicate. Finally, a measured volume of RPMI 1640 was used to suspend the cells, followed by the addition of 10% fetal bovine serum, 10 µg/mL streptomycin and 100 U/mL penicillin and the cell count was adjusted to 2 × 10^6^ cells/mL. From the prepared diluted cell suspension, 0.2 mL was titrated in 96 flat-bottomed tissue culture plates. Serial concentrations of each tested compound were prepared and added in triplicate and incubated at 37 °C for 3 h in a 5% CO_2_ atmosphere. The IC_50_ parameter (half-maximum inhibitory concentration) was calculated using Trypan blue dye exclusion test, and then the cytotoxic effect was evaluated using control EAC cells [39].

### 3.3. 2,2′-Diphenyl-1-picryl-hydrazyl (DPPH) Free Radical Scavenging Activity

To 1 mL of various concentrations of ascorbic acid, neocryptolepine and indoloquinoline analogs prepared from methanol, 1 mL solution of DPPH, 0.1 mM was added. An equal amount of methanol and DPPH served as control. After 20 min incubation in the dark, absorbance was recorded at 517 nm. The experiment was performed in triplicate. The percentage scavenging of the extract was calculated [42,43,44].

### 3.4. Animals

Swiss female albino mice of CD-1 strain (weighing 22 ± 2 gm at the beginning of the experiments) were obtained originally from Schistosome Biological Supply Program (SBSP) unit at Theodor Bilharz Research Institute (TBRI) Giza, Egypt. Mice were caged separately in groups and maintained under standard laboratory conditions, and fed a commercial pelleted diet at parasitological research Lab in Zoology Department, Faculty of Science, Menufiya University, Egypt (Approval No. MNSE2220) and according to the National Institute of Health guide for the care and use of laboratory animals (NIH Publications No.8023, received 1978)

### 3.5. Antitumor Activity

Thalidomide and indoloquinoline derivatives **6a–d** were tested for their antitumor effect; the test was done in vivo on mice-bearing solid tumors. The induction of solid tumor in Swiss female mice performed by inserting 0.2 mL of EAC cells (2 × 10^6^ cell/mL) subcutaneously (SC) between thighs of the lower limb [34]. Seven days after tumor induction, animals were divided into 6 groups (*n* = 10), five groups were injected subcutaneously with 100 mg/kg of tested compounds daily for 5 consecutive days, the sixth group (positive control group) were given 0.3 DMSO/0.7 H_2_O. On day twelve, after carrying out EAC cells, mice were sacrificed, dissected, and change in tumor mass was evaluated by measuring the developed tumor mass volume using the formula [45,46]:

Tumor volume (mm^3^) = 0.52 × length × (width)^2^. (1)

### 3.6. Estimation of Catalase, SOD, and Lipid Peroxidation Levels in Liver Homogenate

The eradicated liver was double washed first, with ice-cold normal saline then, with cold 0.15 mol/L Tris-HCl buffer (pH 7.4). The clean organ was dried and weighed, and used to prepare a 10% *w/v* homogenate in 0.15 mol/L Tris-HCl buffer. A portion of the prepared homogenate was centrifuged at 1500 rpm for 15 min at 4 °C [47] and used for estimating lipid peroxidation, SOD [48] and CAT [49] analysis was carried out using the supernatant.

### 3.7. Immunological Studies

#### 3.7.1. Isolation of Lymphocytes from Spleen

Under sterile conditions, the spleens were eliminated, and the single cell suspension was made in RPMI 1640 medium. Splenic lymphocytes were purified as described earlier [50]. Viable splenic lymphocytes were counted in a hemocytometer by trypan blue exclusion test and used for further analysis.

#### 3.7.2. Trypan Blue Exclusion Test of Cell Viability

During the study, female Swiss albino mice (normal, treated, and untreated) were anesthetized. Under sterile conditions, after scattering 70% alcohol on the abdominal region, the spleen was removed and then injected with a minor amount of PBS. Spleen was pressed across the fine mesh of the tissue grinder. After centrifuging the produced cell suspension at 1000 rpm for 5 min, the supernatant was discarded, and the cells were washed at room temperature by centrifugation in PBS twice. Trypan blue extrusion method was used to evaluate cell viability, and a phase-contrast microscope was used for cell counting. The experiment was done in tripartite [51].

#### 3.7.3. Lymphoproliferative to Phytohemagglutinin (PHA) Assay

The lymphoproliferative assay was done according to a reported method [52]. In brief, lymphocytes count was adjusted to 1 × 10^6^ cells/mL with complete RPMI-1640. 100 µL of cell suspension and was dispensed into each well of a 96-well plate. Equal volumes of PHA (µg/mL) was added to lymphocytes in triplicate. The plate was incubated for 3 days in a humidified 37 °C, 5% CO_2_ incubator. 5 µL of [^3^H] thymidine (50 µ Ci/mL) was placed into each well 18 h prior to the end of incubation. At the end of incubation, cells were harvested by using a cell harvester apparatus and were counted in ß counter. The count per minute (cpm) for each well was determined, and the stimulation index (SI) for lymphocytes was calculated according to the equation: SI = cpm (test)/cpm (normal)

### 3.8. Flow Cytometric Analysis of Compounds Effects on EAC Cells Cell Cycle Distribution

Fresh tumor tissue specimens from treated and untreated groups were transported to our laboratory in isotonic saline. The specimens were prepared following these steps:

1. The material was washed with isotone tris EDTA buffer. Then, the cell suspension was centrifuged at 1800 rpm for 10 min; the supernatant was then separated;
2. The cell is then fixed in ice-cold 96–100% ethanol (BDH) in approximately 1 mL for each sample;
3. Two milliliters of phosphate buffer solution (PBS) were used to wash the sample, which was recentrifuged at 1500 rpm for 5 min then the supernatant was thrown away;
4. Granules of the Tumor cells were put back into suspension using 200–500 µL of PBS. Into polystyrene tube, 100 µL of the suspension was transferred and then stained with 1.5 mL of propidium iodide. The tube is kept at 4 °C in a dark place for one hour;
5. The sample was run in the flow cytometer within 30 min after the addition of propidium iodide.

The flow cytometer was carried out with FACS caliber flow cytometer (Becton Dickinson, Sunnyvale, CA, USA) equipped with a compact air cocked low-power 15 m watt argon ion laser beam (488 nm). The average number of evaluated nuclei per specimen 20,000, and the number of nuclei scanned was 120 per second. DNA histogram derived from flow cytometry was obtained with a computer program for Dean and Jett’s mathematical analysis. Data analysis was conducted using DNA analysis program MPDFIT (Verity Software House, Inc. Box 247, Topsham, ME 04086 USA, version: 2.0, power Mac with 131,072 KB Registration No.: 42000960827-16193213 Date made: 16 September 1996). This software calculated the CV around the G0/G1 peak and the percentage of cells in each phase (G0/G1, S and G2/M) of the DNA cell cycle for each sample.

### 3.9. Histopathologic Examination

Each tumor or liver sample for each mouse was separated and kept in 10% formalin solution and dehydrated in a graded alcohol series. Each specimen was treated with xylene and then inserted in paraffin blocks. The specimen was cut into sections, the thickness of each section is five-micron, hematoxylin, and eosin (H and E) were added for staining. The stained liver specimen was used to assess the degree of degradation and necrosis using a reported scaling system [53]; the scaling system use numbers from 0 to 3 to reflect necrosis severity. Starting from 0, which indicates no necrosis and moves to mild, moderate, and severe necrosis indicated by 1, 2, and 3, respectively. Mild and moderate stages are defined as focal and multifocal necrosis or degradation of hepatocytes, respectively, while severe is characterized by local immense, spread out necrosis or damaged liver cells. Tumor specimen stained with H and E were used to evaluate the severity and percentage of necrosis by the same previously mentioned scaling system [54].

H and E-stained slides of tumor specimens were used also used to calculate the H score [55] as well as the mitosis counting [56] and apoptotic tumor cell counting [57,58], ten arbitrarily chosen microscopic fields were used for each mitosis and apoptotic tumor cell counting, this was considered as examining at least 1000 tumor cells. High magnification (400×) was used for microscopic examination.

## 4. Conclusions

In the present study, four neocryptolepine analogs were synthesized and evaluated for their anticancer potential in Ehrlich ascites.

Carcinoma-induced solid tumor in Swiss albino mice. Based on in vitro studies such as cytotoxicity, antioxidant, and in vivo studies in animal models such as evaluation of tumor volume and endogenous antioxidant activity, we can conclude that neocryptolepine was confirmed as a useful lead compound for the development of new anticancer agents. In addition, the histopathological investigations revealed that the tested neocryptolepine have necrotic, antimitotic, and apoptotic activities against solid tumors with minimum side-effect on the liver. Our initial goal to prepare synthetic derivatives with higher anticancer activity could be achieved, resulting in analogs with in vitro and in vivo activity. Further variations in substituents and substitution patterns may be necessary to obtain more potent analogs showing in vivo activity with a higher margin of safety.

## Figures and Tables

**Table 1:** In vitro cytotoxic activities of indoloquinoline analogs 6a–d versus the reference drug, thalidomide and the parent natural compound, neocryptolepine.

Groups	IC_50_ (µM)
Thalidomide	2.6 × 10^−4^ ± 0.76
Neocryptolepine	5.4 × 10^−4^ ± 0.65
**6a**	1.7 × 10^−4^ ± 0.723
**6b**	6.4 × 10^−5^ ± 0.76
**6c**	3.3 × 10^−4^ ± 0.82
**6d**	1.5 × 10^−4^ ± 0.86

**Table 2:** Determination of the in vitro antioxidant activities of indoloquinoline analogs using 2, 2-diphenyl-1-picrylhydrazyl (DPPH) photometric assay.

Groups.	DPPH IC_50_ (µM) ^1,2^
Ascorbic acid	4.2 × 10^−5^ ± 1.49
Neocryptolepine	1.6 × 10^−4^ ± 0.50
**6a**	4.7 × 10^−5^ ± 4.26
**6b**	4.2 × 10^−5^ ± 3.67
**6c**	9.2 × 10^−5^ ± 2.16
**6d**	3.3 × 10^−5^ ± 3.75

^1^ Results are (means ± SD) (*n* = 3). ^2^ IC_50_: The amount of compound needed to scavenge 50% of DPPH radicals.

**Table 3:** Percentage mortality in Swiss albino mice given indoloquinoline analog **6d** (subcutaneously (SC)) at different doses.

Groups	No. of Animals	Dose (mg/kg)	No. of Death	% Mortality
1	7	400	0	0
2	7	800	2	28.5
3	7	1200	4	57.1
4	7	1600	6	85.7
5	7	2000	7	100
6	7	DMSO:saline (0.3/0.7)	0	100

**Table 4:** Effect of tested indoloquinoline derivatives on lipid peroxidation and antioxidant enzymes in normal mice.

Groups	Lipid Peroxidation (nmol MDA/g Tissue)	SOD (U/g Tissue)	CAT (KU/mg Tissue/s)
Normal	249 ± 1.6	139.96 ± 0.86	7.05 ± 0.18
**6a**	252.1 ± 7.2	139.4 ± 2.9	6.5 ± 0.39
**6b**	247.96 ± 6.3	140.2 ± 1.9	5.7 ± 0.31
**6c**	251.68 ± 11.9	138.6 ± 6.0	6.2 ± 0.30
**6d**	248.69 ± 5.5	137.9 ± 3.9	6.1 ± 0.20

**Table 5:** Effect of tested indoloquinoline derivatives on lipid peroxidation and antioxidant enzymes in solid-tumor bearing mice.

Groups	Lipid Peroxidation (nmol MDA/g Tissue)	SOD (U/g Tissue)	CAT (KU/mg Tissue/s)
Normal	249 ± 1.6	139.96 ± 0.86	7.05 ± 0.18
Positive control	413.4 ± 6.2	95.03 ± 3.73	4.1 ± 0.13
Thalidomide	330.2 ± 8.1	108.4 ± 10.2	5.8 ± 1.5
**6a**	249.3 ± 2.1 *	146.75 ± 2.11 *	6.2 ± 0.14 *
**6b**	268.9 ± 2.8 *	146.06 ± 3.11 *	7.3 ± 0.16 *
**6c**	262.1 ± 1.9 *	129.47 ± 1.38 *	8.5 ± 0.34 *
**6d**	249.9 ± 2.2 *	132.86 ± 0.55 *	6.8 ± 0.12 *

* Statistical significant change as compared with positive control group (*p* < 0.001).

**Table 6:** Effect of indoloquinoline derivatives on splenic lymphocyte number in the spleen of normal mice.

Groups	Splenic Lymphocyte Number (10^6^/mL)
Normal control	14.7 ± 1.26
**6a**	13.3 ± 1.08
**6b**	12.97 ± 0.65
**6c**	13.29 ± 1.12
**6d**	12.8 ± 0.04

**Table 7:** Effect of indoloquinoline derivatives on splenic lymphocyte number in the spleen of EAC bearing mice.

Groups	Splenic Lymphocyte Number (10^6^/mL)
Normal control	14.7 ± 1.26
Positive control	3.7 ± 0.43 *
**6a**	20.0 ± 0.73 *
**6b**	14.8 ± 1.22 *
**6c**	17.0 ± 0.58 *
**6d**	17.0 ± 0.61 *

* Statistical significant change as compared with normal control group (*p* < 0.001).

**Table 8:** Lymphoproliferative response to phytohemagglutinin mitogen (cpm) in control and EAC bearing mice treated with indoloquinoline derivatives.

Groups	PHA Lymphoproliferative (cpm)
Normal control	2.4 ± 0.15
Positive control	0.44 ± 0.07
**6a**	2.4 ± 0.1 *
**6b**	1.8 ± 0.1 *
**6c**	2.3 ± 0.14 *
**6d**	2.3 ± 0.14 *

* Statistical significant change as compared with normal control group (*p* < 0.001).

**Table 9:** Lymphoproliferative response to phytohemagglutinin mitogen (cpm) in normal control and indoloquinoline derivatives bearing mice.

Groups	PHA Lymphoproliferative (cpm)
Normal control	2.4 ± 0.15
**6a**	2.4 ± 0.11
**6b**	2.2 ± 0.21
**6c**	2.3 ± 0.09
**6d**	2.3 ± 0.12

**Table 10:** Flow cytometric analysis of cell cycle distribution of EAC cells.

Groups	Apoptotic Cells (sub-G1)	Non-Apoptotic Cells		
		G0/G1	S	G2/M
Positive control	1.57 ± 0.02	76.87 ± 4.17	7.92 ± 0.43	15.21 ± 0.6
**6a**	3.23 ± 0.01 *	90.27 ± 6.35 *	5.12 ± 0.36 *	4.61 ± 0.6 *
**6b**	24.36 ± 0.01 ***	78.44 ± 0.04 *	2.35 ± 0.13 *	19.21 ± 0.09 *
**6c**	12.30 ± 0.15 ***	3.30 ± 0.15 ***	40.50 ± 2.41 ***	56.20 ± 0.1 ***
**6d**	16.88 ± 0.6 ***	41.30 ± 0.31 *	3.44 ± 0.27 *	55.26 ± 0.06 ***

* Statistical significant change as compared with positive control group (*p* < 0.05). *** Statistical significant change as compared with positive control group (*p* < 0.001).

## Data Availability

The data presented in this study are available on request from the corresponding author.

## Supplementary Materials

Supplementary File 1

## Sample Availability

Samples of the compounds are available on request from the authors.
