# Supplementary material for: In Vitro and In Vivo Antitumor Activity of Indolo[2,3-b] Quinolines, Natural Product Analogs from Neocryptolepine Alkaloid
Source: Molecules. 2021 Feb 1;26(3):754. doi: 10.3390/molecules26030754 (PMC7867085; doi:10.3390/molecules26030754)

Sample No. 6a

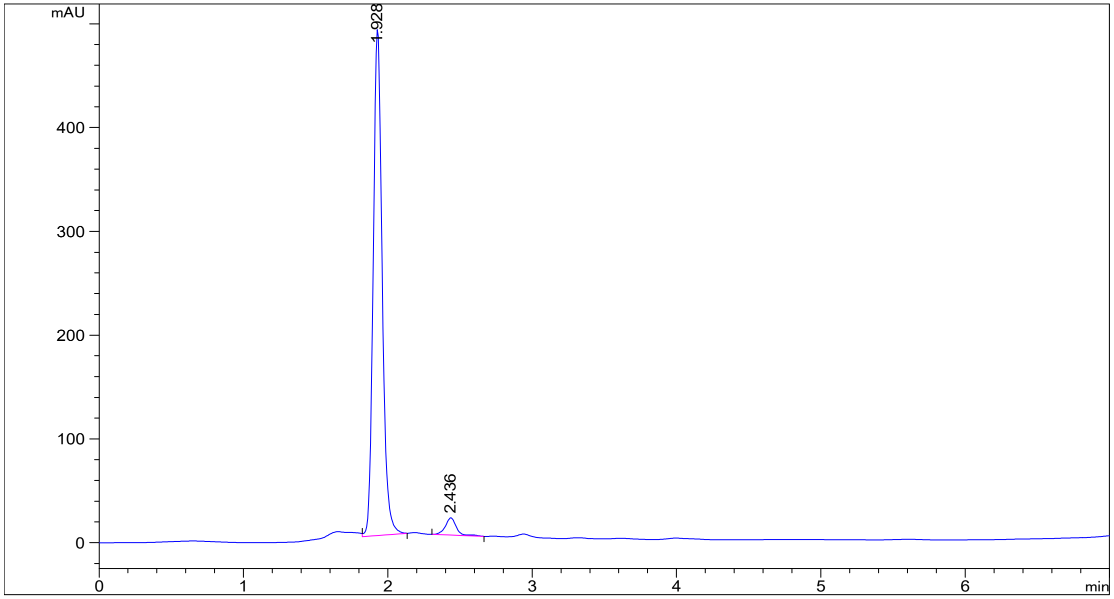

#	Time	Area	Height	Width	**Area%**	Symmetry
1	1.928	2068.9	486.7	0.0656	**95.894**	0.834
2	2.436	88.6	16.6	0.0784	**4.106**	0.929

Sample No. 6b

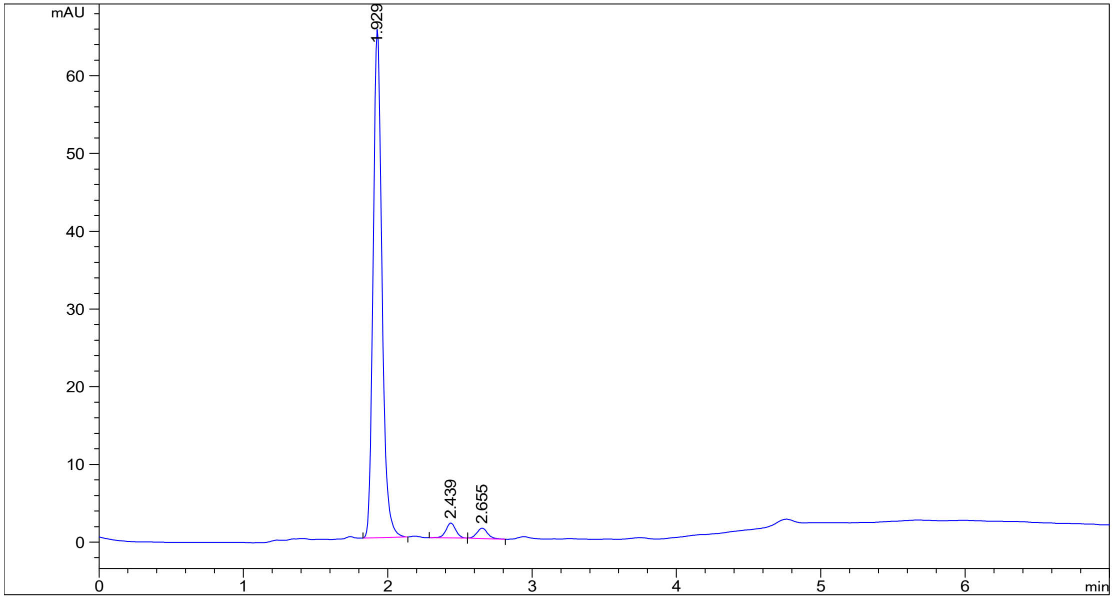

#		Time	Area	Height	Width	Area%	Symmetry
	1	1.929	278.7	65.5	0.0637	**94.43**	0.815
	2	2.439	9.3	1.9	0.072	**3.141**	0.932
	3	2.655	7.2	1.4	0.0796	**2.429**	0.926

Sample No.6c

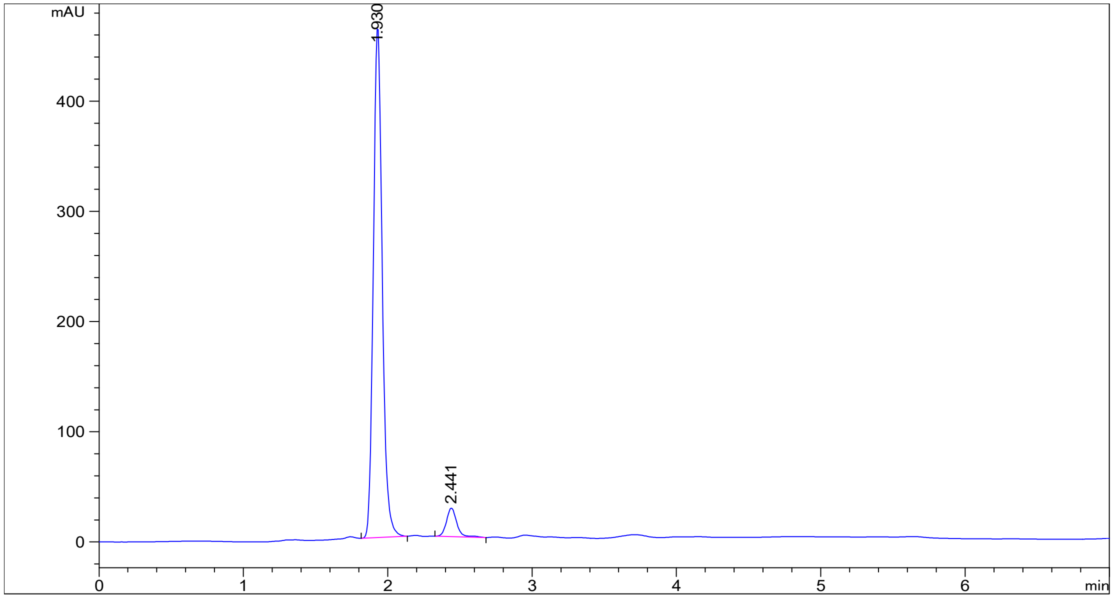

#	Time	Area	Height	Width	**Area%**	Symmetry
1	1.93	1983.4	464.6	0.0658	**93.87**	0.848
2	2.441	129.5	26	0.0761	**6.13**	0.831

Sample No. 6d

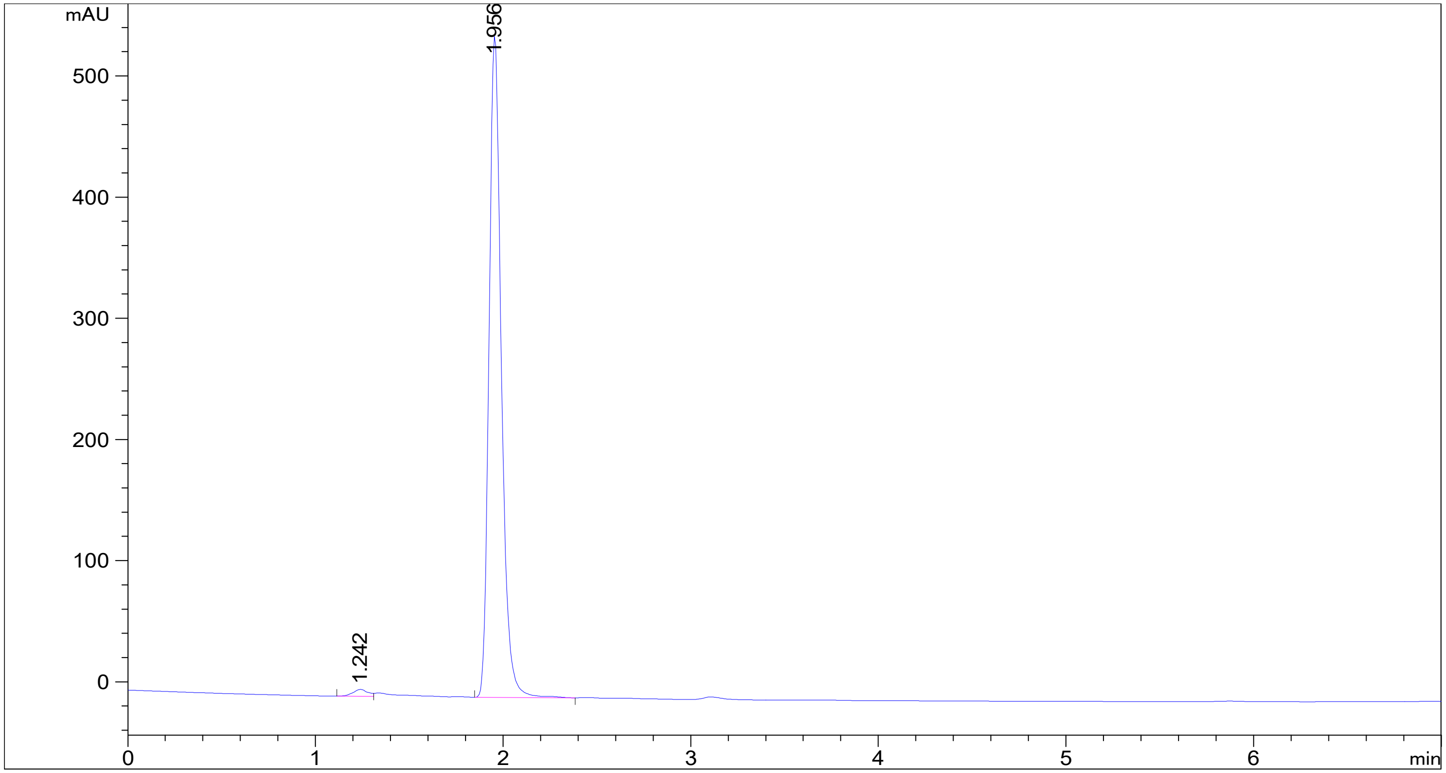

#	Time	Area	Height	Width	**Area%**	Symmetry
1	1.242	32	5.8	0.0803	**1.271**	0.989
2	1.956	2487.9	546.3	0.0711	**98.729**	0.758

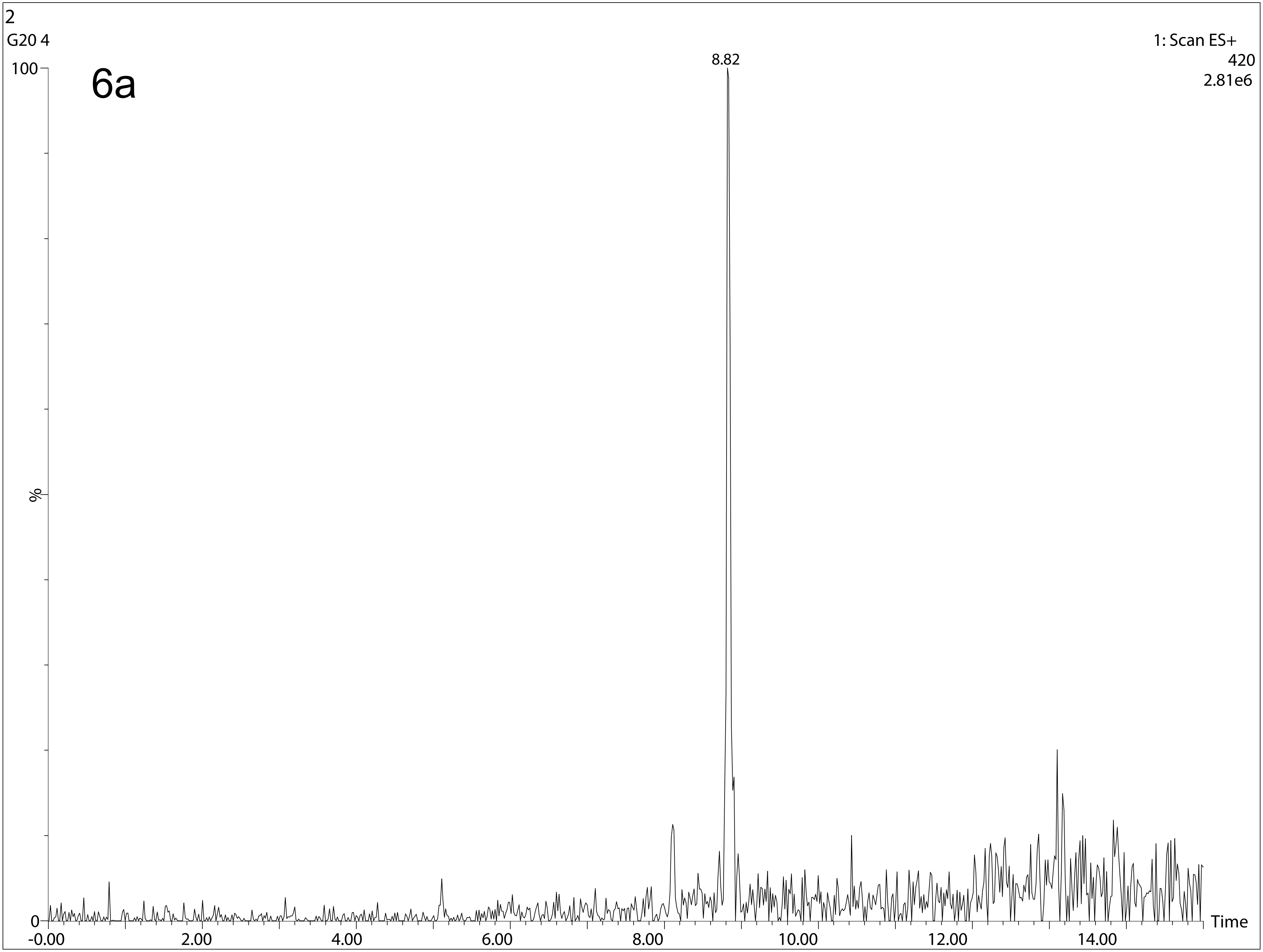
2
G20 4
6a
1: Scan ES+
420
2.81e6
8.82
100
%
0
-0.00
2.00
4.00
6.00
8.00
10.00
12.00
14.00
Time

Openlynx Report -

Sample: 440 Vial:1:A,2 ID:
File:G20 4DS Date:05-Jan-2021 Time:11:51:55
Description:2

Printed: Tue Jan 05 12:44:38 2021

Peak ID	Time	Error PPM
13	8.28	

(Time: 8.28)

6a

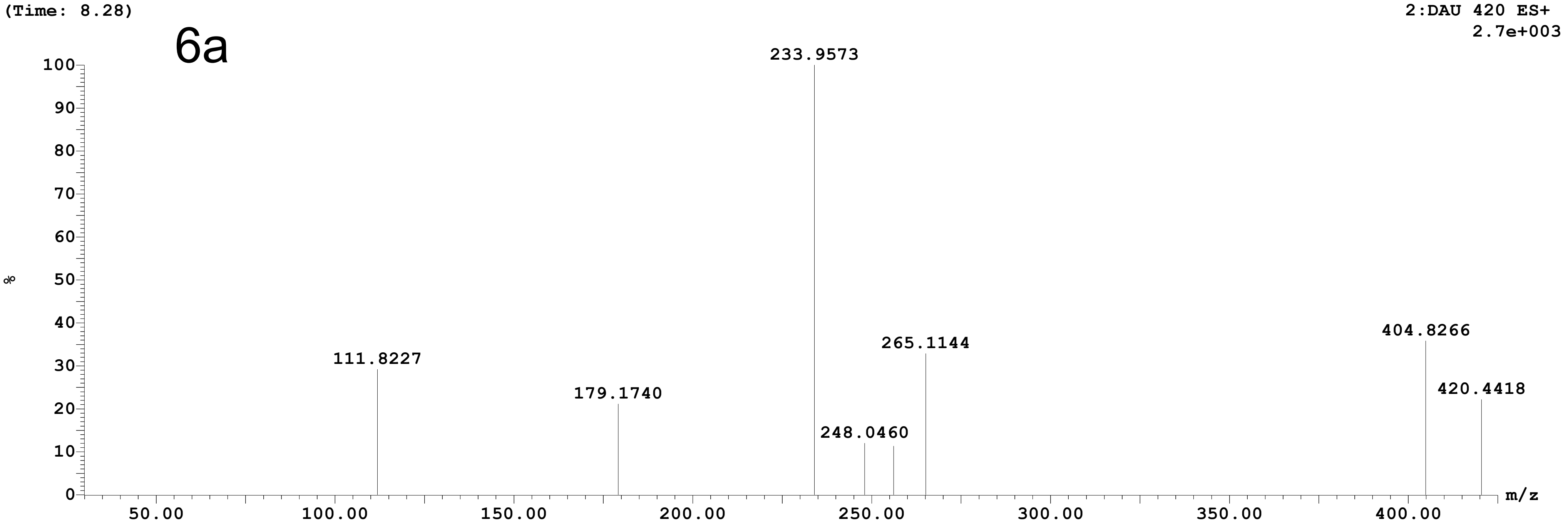

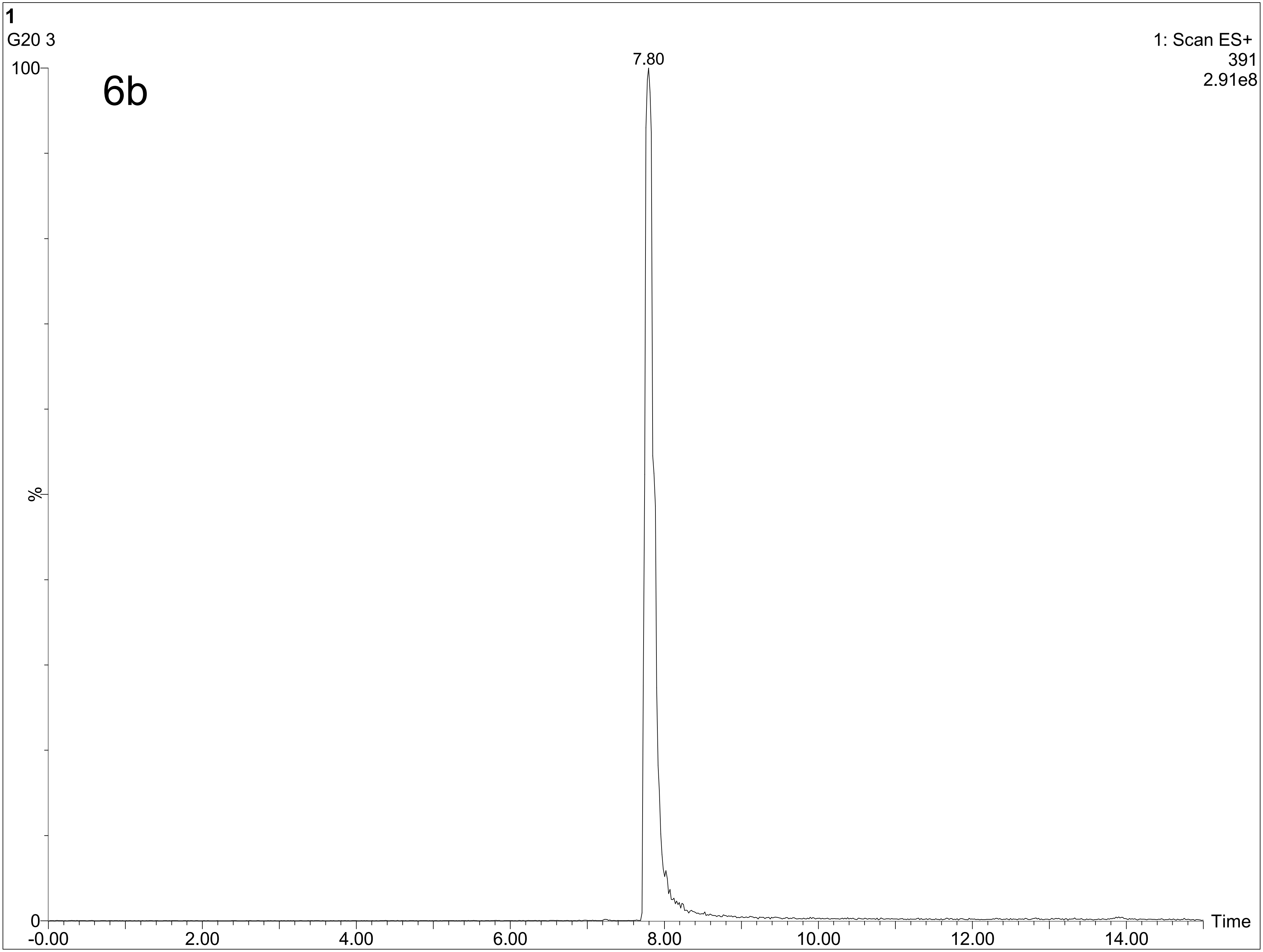

1
G20 3
6b
1: Scan ES+
391
2.91e8
7.80
100
%
0
-0.00
2.00
4.00
6.00
8.00
10.00
12.00
14.00
Time

Center for DRUG DISCOVERY RESEARCH and DEVELOPMENT
Openlynx Report -

Sample: 439 | Vial:1:A,1 | ID:
File:G20 3DS | Date:05-Jan-2021 | Time:11:36:06
Description:1

Printed: Tue Jan 05 12:44:25 2021

Peak ID	Time	Error PPM
4	8.07	

(Time: 8.07)

6b

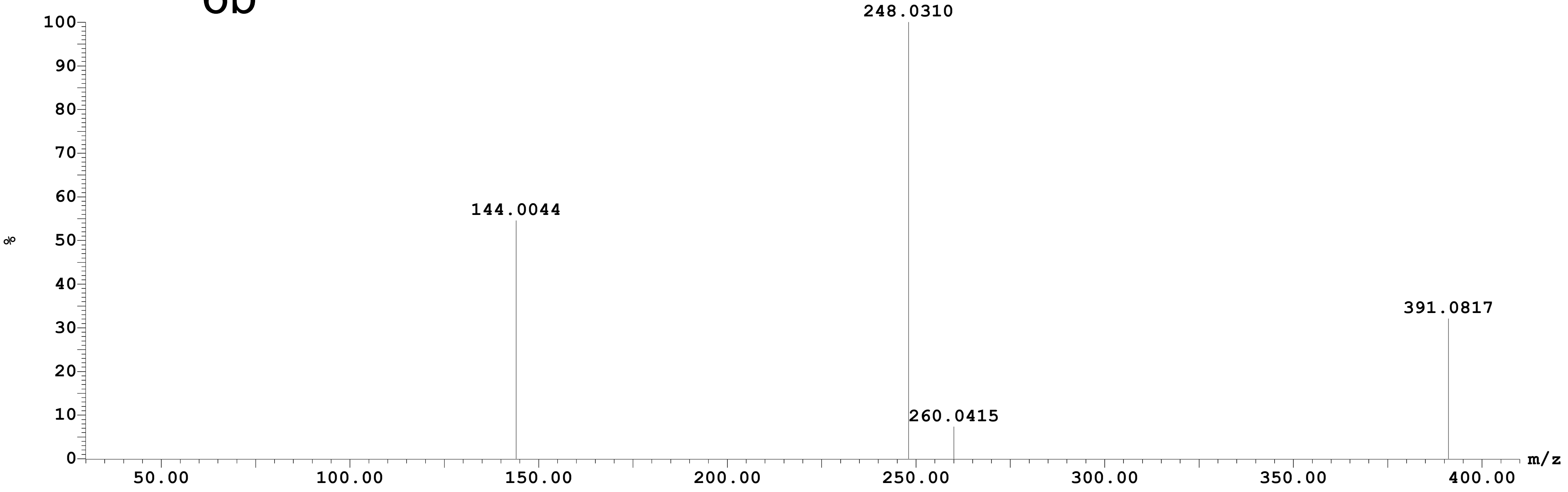

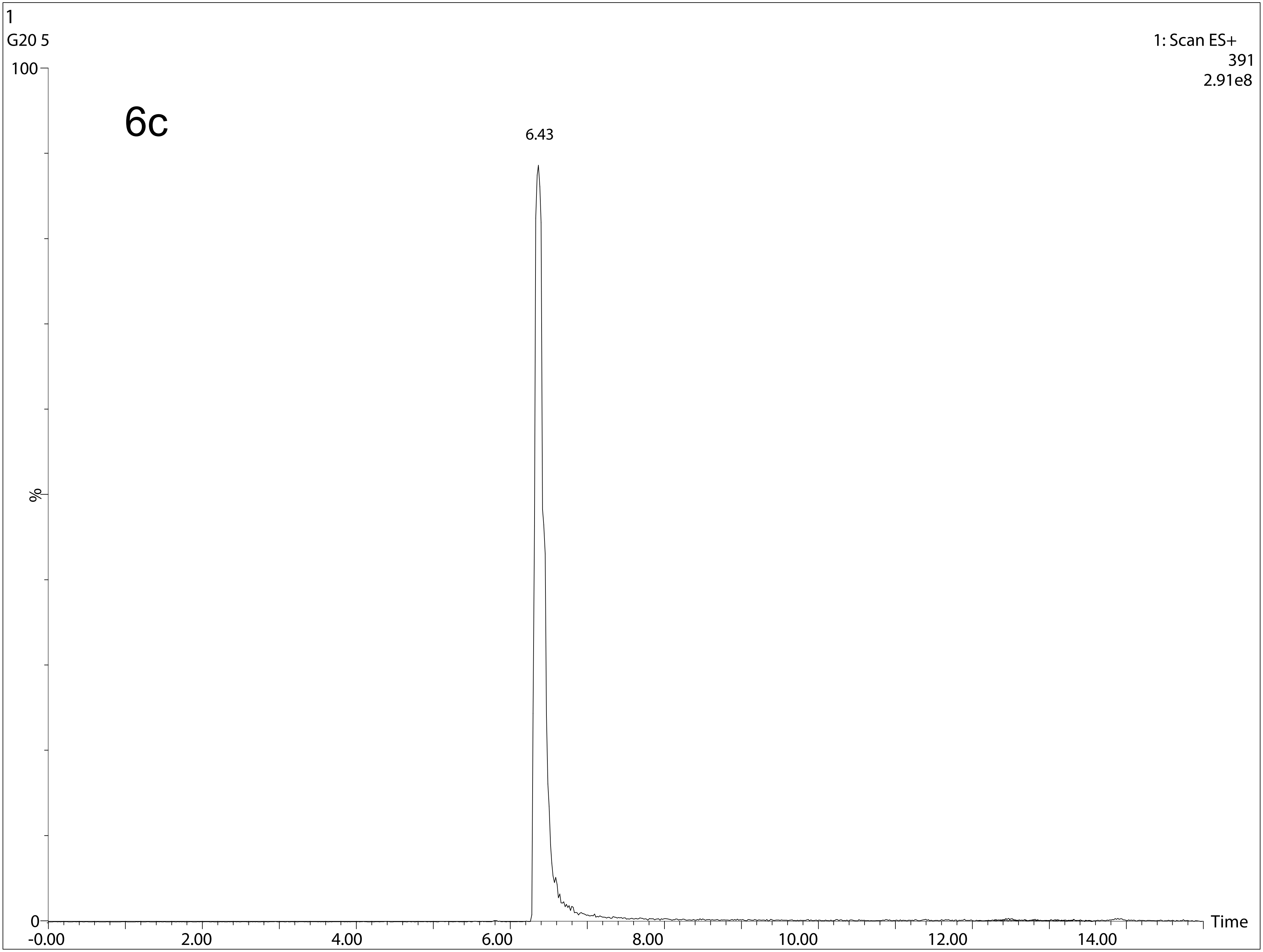
1
G20 5
1: Scan ES+
391
2.91e8
100
6c
6.43
%
0
Time
-0.00
2.00
4.00
6.00
8.00
10.00
12.00
14.00

Openlynx Report -

Sample: 441 | Vial:1:A,3 | ID:
File:G20 5DS | Date:05-Jan-2021 | Time:12:07:50
Description:3

Printed: Tue Jan 05 12:44:53 2021

Peak ID	Time	Error PPM
18	6.71	

(Time: 6.71)

6c

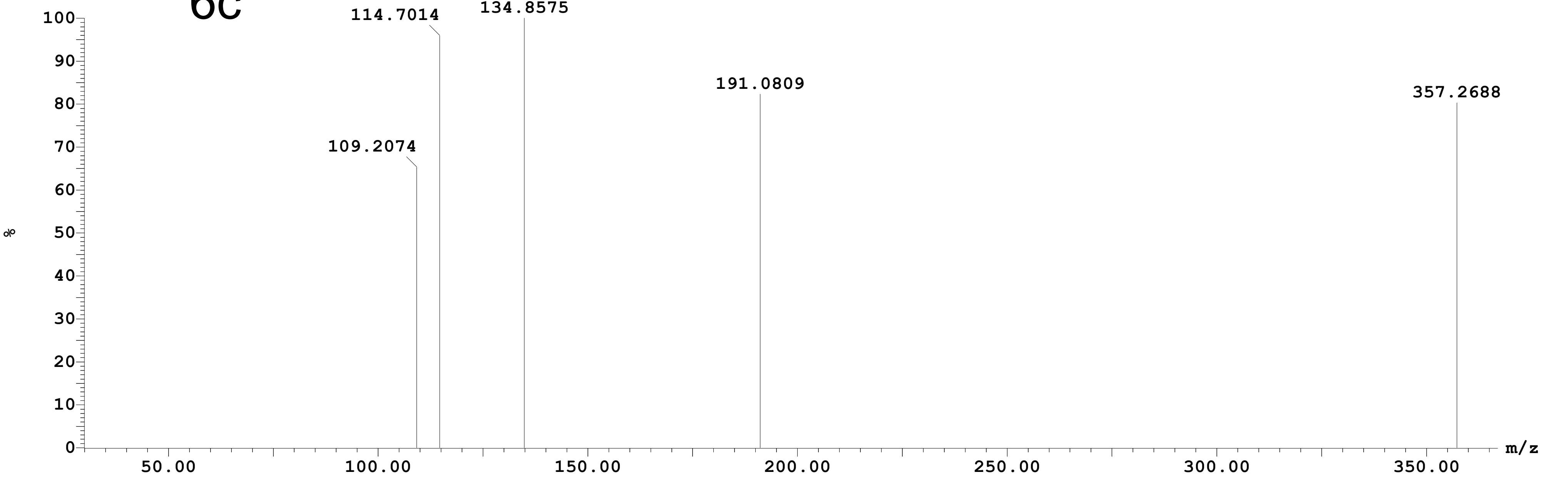

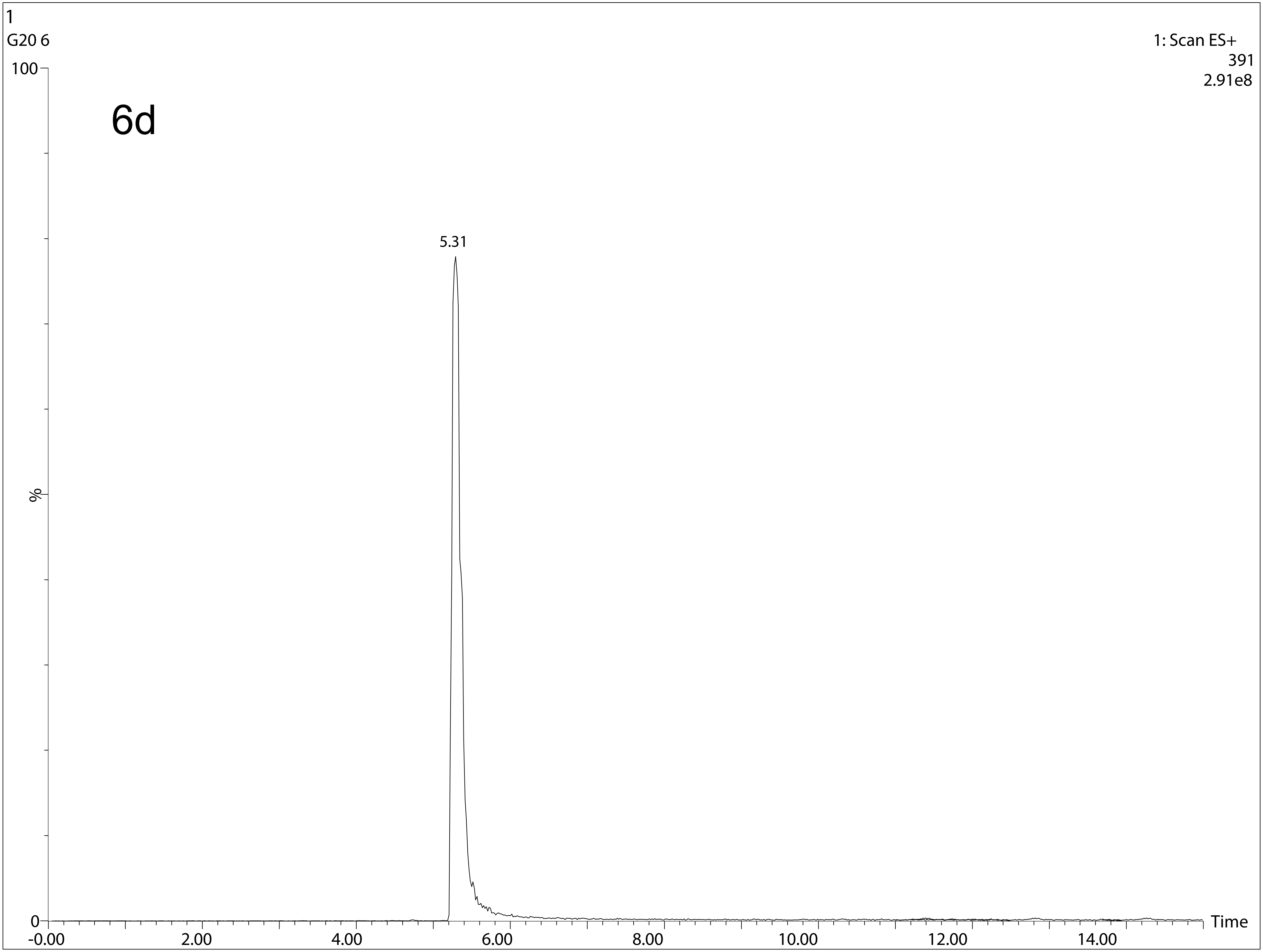

1
G20 6
1: Scan ES+
391
2.91e8
100
6d
5.31
%
0
Time
-0.00
2.00
4.00
6.00
8.00
10.00
12.00
14.00

Center for DRUG DISCOVERY RESEARCH and DEVELOPMENT
Openlynx Report -

Sample: 442	Vial:1:A,4	ID:
File:G20 6DS	Date:05-Jan-2021	Time:12:23:39
Description:4		

Printed: Tue Jan 05 12:45:03 2021

Peak ID	Time	Error PPM
5	3.30	

6d

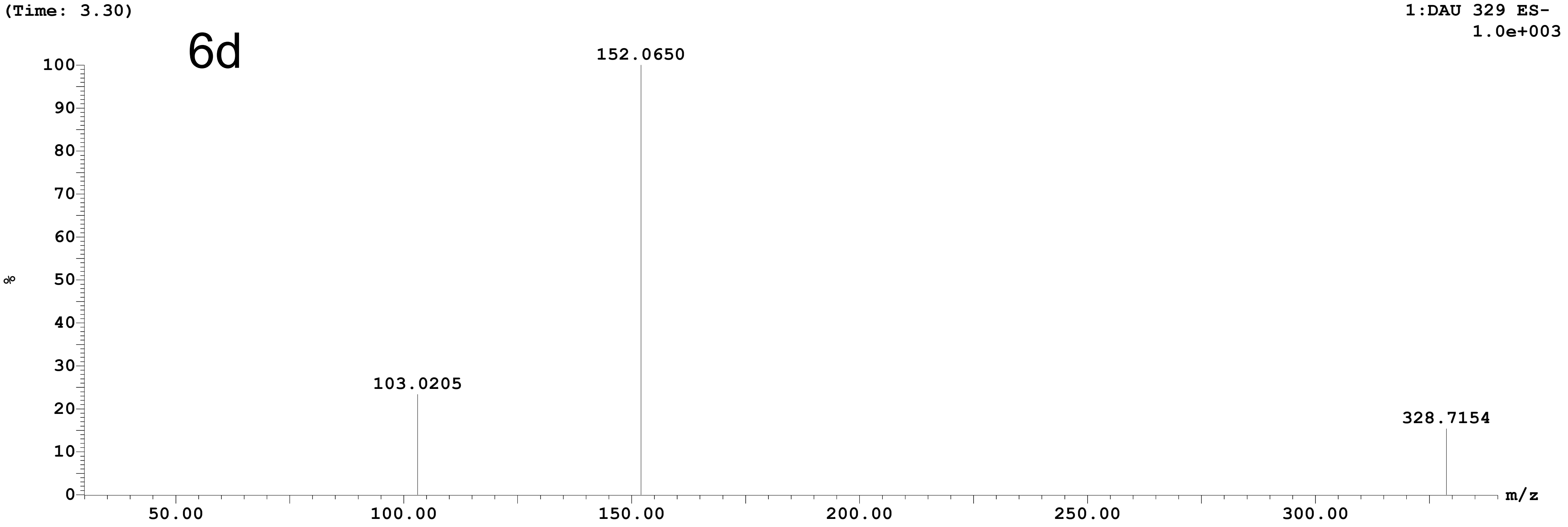

Supplement: Supplementary File 1 [file molecules-26-00754-s001.pdf]
